# Food Behaviors, Health, and Bean Nutrition Awareness among Low-Income Men: A Pilot Study

**DOI:** 10.3390/ijerph17031039

**Published:** 2020-02-06

**Authors:** Michelle M. Heer, Donna M. Winham

**Affiliations:** Food Science & Human Nutrition, Iowa State University, Ames, IA 50010, USA; mmheer@gmail.com

**Keywords:** food behavior, nutrition knowledge, pulses, purchasing preferences, health disparities

## Abstract

Bean consumption is known to lower blood cholesterol and postprandial blood glucose. With higher chronic disease risk, low-income men could theoretically benefit from increased bean intakes. The study objective was to explore low-income men’s food behaviors, bean health benefit awareness, and bean consumption practices and preferences. Seventy-one low-income men aged 18–65 years (µ 41 ± 12.7; 53% white, 16% black, 31% Hispanic) completed a survey on health risks, food behaviors, bean health knowledge, attitudes toward dry and canned beans, and bean preferences. Eighty-seven percent had one or more health risk factors of physical inactivity, smoking, or obesity. Most men compared food prices, and thought about healthy food choices for their families, but few planned meals or read nutrition facts labels. White men had significantly higher bean health benefit knowledge than black or Hispanic men (*p* < 0.01). Most men liked the taste of beans, disagreed dry beans took too long to prepare, and 79% ate them at least 2–3 times per month. Forty-nine percent agreed beans caused intestinal gas. Improving men’s awareness of the health benefits of beans as well as leveraging existing positive attitudes may be useful approaches to increase bean consumption among low-income and minority male populations.

## 1. Introduction

The leading causes of death among adult men in the United States (US) remain heart disease (24.4%) and cancer (21.9%) [1]. The risk factors for these diseases as well as type 2 diabetes, hypertension, and obesity are mediated by lifestyle behaviors such as diet, physical activity, regular medical checkups, and not smoking [2]. Men may be less concerned about healthy dietary patterns and lack awareness of diet-health associations in comparison to women [3].

Along with gender, wage earnings and race/ethnicity have a considerable impact on health and disease outcomes [4]. Low-income men have increased prevalence of health risk factors including physical inactivity, smoking, and obesity, as well as increased chronic disease rates such as heart disease, stroke, diabetes, and cancer [4,5]. Disease rates are greater in blacks and Hispanics than non-Hispanic whites, yet within race/ethnicity groups, rates are linked inversely to income [1,4,5].

Not unexpectedly, low-quality diets are associated with limited economic resources [6,7]. A lack of dietary diversity accompanied with high levels of saturated fat and low vegetable, fruit, and fiber intake contributes to disease risk [4]. Pulses, which include beans, peas, lentils and chickpeas, can improve dietary quality by providing protein, fiber, and many micronutrients associated with other vegetables, including folate, iron, zinc, magnesium, and potassium [8,9]. Compared to other foods, beans have one of the best nutrient-to-price ratios, making them ideal for maximizing nutrition with economic constraints [9]. Bean consumption is associated with both reduced risk and improved outcomes of cardiovascular disease [10], serum cholesterol [11], postprandial glucose control [12], type 2 diabetes [13], and some cancers [14]. The dietary fiber in beans is likely a contributing component to lower risk of these chronic diseases [15].

The 2015–2020 Dietary Guidelines for Americans (DGA), which aim to promote health, prevent chronic disease, and help Americans maintain a healthy weight, suggests 2–3 cups of cooked beans per week for men ages 19–50 [2]. On average, men currently consume 1 cup per week [2]. Increasing bean consumption to meet the DGA could improve nutrient shortfalls such as fiber, potassium, and iron, and reduce disease-related risks [15,16]. Only a handful of published studies exist on the factors that influence bean consumption among Americans, and these are mostly with minorities or women [14,17,18,19,20,21]. Determining the views and current knowledge toward beans among low-income males is necessary to design tailored intervention programs to boost bean intake and improve health.

There were five study objectives. These were to determine low-income men’s: (1) health risk factors; (2) food behavior practices; (3) knowledge of bean health benefits; (4) attitudes and perceptions toward dry and canned beans; and (5) consumption patterns and purchasing preferences of beans.

## 2. Materials and Methods

### 2.1. Participants and Data Collection

Low-income male clients, aged 18–65 years old, were recruited from an employment center, a Special Supplemental Nutrition Program for Women, Infants and Children (WIC) clinic, and classes of the Expanded Food and Nutrition Education Program (EFNEP). With agency permission, researchers set up an information table in the waiting rooms of the employment center and WIC. Signage and flyers promoted a survey on health and food behaviors, but did not specifically mention beans to reduce response bias to bean consumers only. If a man expressed interest in participating, a description of the study was read to him, and verbal consent was given before he received the survey in English or Spanish based on his choice. Participants received a $3 cash incentive at the employment center and WIC. At the EFNEP classes, men completed surveys at the end of the third session of the six-class series. Men received grocery coupons and brand-marketed notepads, pens, tote bags, etc. Data were collected in March through September 2011. The Arizona State University Institutional Review Board deemed the study exempt [#1009005462].

### 2.2. Survey Instruments

The survey consisted of six parts: demographics, health risk factors, food behaviors, food security, attitudes and perceptions of dry and canned beans, and bean purchasing preferences. The demographic questions and the 10-item Food Behavior Checklist from the Arizona EFNEP program enrollment form were used for all sites [22,23]. Health behavior questions (smoking, physical activity, health care) were from the Behavioral Risk Factor Surveillance System (BRFSS) [24]. The six-item USDA Core Food Security Module was used to evaluate food security status [25]. Dry and canned bean attitude and perceptions Likert statements, and bean purchasing preferences were adapted from previous research [17,19].

Prior to data collection, the draft survey content was reviewed with seven Extension instructional specialists and subsequently pilot tested with two focus groups of EFNEP participants (n = 24). After modifications from this formative evaluation, 11 more agency researchers and field staff evaluated the survey for content validity. Another group of 23 EFNEP participants completed the survey and gave post-test feedback before formal data collection began [26,27].

### 2.3. Statistical Analysis 

BMIs were calculated from self-reported height and weight. These raw values were classified into categories of normal, overweight, and obese [28]. Descriptive statistics were compared by race/ethnicity categories using Chi-square and ANOVA. Likert statement responses for dry and canned bean were condensed from five to three categories of ‘disagree, neutral, agree’ for display in tables. Principal components analysis was applied to the five-category Likert-type responses for four groups of questions (the 10-item Food Behavior Checklist, health benefits of beans, attitudes toward dry beans, and attitudes toward canned beans) to identify thematic clustering. Reliability analysis to develop parsimonious models resulted in four scales with good reliability (Cronbach’s alpha in parentheses): positive food behaviors (0.74), bean health knowledge (0.86), general bean attitudes (0.78) and canned bean attitudes (0.76) [29]. Data entry and analysis was performed with SPSS V.23.0 (IBM, Armonk, NY, USA), and a *p* value <0.05 was considered significant.

## 3. Results

The completion rate was 91.5% (75/82). Four men (2 Native American, 2 Asian) were excluded from further analysis due to small sample size for ethnic comparisons. Sixty percent of the respondents were employment center clients, with 22% recruited from WIC and 13% from EFNEP. Over half of the men (53%) identified as white, 16% as black, and 31% as Hispanic. Hispanic men were younger (*p* = 0.021), had fewer years of education (*p* = 0.009), more children in the household (*p* = 0.001), and a greater percentage were born outside of the US (*p* < 0.001) than their peers. Eighty-seven percent of all men had one or more risk factors of physical inactivity, smoking, or obesity. There were no significant differences by race/ethnicity for food security, body mass index (BMI), exercise frequency, smoking, health care coverage, or routine checkup frequency (Table 1).

Table 2 shows the frequency responses to the Food Behavior Checklist statements on food resource management, food safety, and nutrition practices by race/ethnicity [17]. Seventy-three percent of the men compared prices before buying food, but black and Hispanic men did this less often (*p* = 0.046). Higher percentages of black and Hispanic men reported running out of food by the end of the month than white men (*p* = 0.001). While 70% of all men said they did not leave meat or dairy out of the refrigerator for more than 2 hours, over 60% of black and Hispanic men said they thawed foods at room temperature (*p* = 0.028). Hispanic men planned meals ahead of time ‘sometimes’ as opposed to ‘always’ in contrast to their peers (*p* = 0.039). The ‘positive food behavior’ scale mean was higher for white men, suggesting a trend of more frequent behaviors, than among black or Hispanic men (*p* = 0.056).

Responses to Likert-type statements on the health benefits of beans are provided in Table 3. Close to 60% of all men agreed beans improve nutrition but about 25% of black and Hispanic men disagreed (*p* = 0.027). Almost 50% of all men stated a ‘neutral’ response regarding the ability of eating beans to lower cholesterol, lower cancer risk, or control blood sugar. Significantly more black and Hispanic men disagreed that eating beans could ‘lower bad cholesterol’ (*p* = 0.016), control blood sugar (*p* = 0.049), or are healthy for the gastrointestinal tract (*p* = 0.044). The bean health benefits scale was significantly higher for white men indicating more knowledge than black or Hispanic men (*p* = 0.008).

The Likert-type statements for attitudes and perceptions of dry and canned beans are shown in Table 4. Over 70% of men disagreed it is difficult to make food with beans and that only ‘poor people’ eat beans. Few men agreed that they disliked the taste of beans, that their family will not eat beans, or that their friends do not eat beans. Almost half of the men agreed beans cause intestinal gas. Only 32% agreed that dry beans take too long to prepare. Similar positive attitudes were expressed by most men for the seven canned bean statements. There were no significant differences by race/ethnicity for the statements or the general bean or canned bean scale scores.

Men’s bean consumption, purchasing practices, and preferences are shown in Table 5. Significantly more black and Hispanic men purchased dry beans only (*p* = 0.050) compared to white men. Of the men who purchased beans, more white than black or Hispanic men indicated price was an important selection characteristic for dry beans (*p* = 0.036) and for canned (*p* = 0.017). Over 70% of Hispanic men who bought dry beans stated that they selected a dry bean based on tradition (*p* = 0.003) in comparison to their peers. Men were asked how often they consumed eight bean types.

Sixty-three percent stated they ate pintos more than once per month, followed by baked (31%), black (27%), dark red kidney (25%), and chickpeas (21%). Fewer than 15% of men reported eating navy, lentils, or black eyed peas more than once per month (data not shown). Of the 78% of men who reported buying canned beans, 36% did not purchase a specific brand, 20% did not remember the brand name, 17% bought Bush Brothers & Company (Knoxville, TN, USA) and 14% mentioned Rosarita (Conagra, Chicago, IL, USA) (data not shown).

## 4. Discussion

The purpose of the current study was to explore the food behaviors, knowledge, and attitudes toward dry and canned beans among a sample of 71 low-income men. Comprehensive details were collected on health risk factors, food behavior practices, knowledge of the health benefits of beans, attitudes and perceptions toward dry and canned beans, and consumption patterns and purchasing preferences of beans.

Most men had one or more health risk factors of physical inactivity, current smoking or obesity. In comparison to other men in Arizona, study participants were physically active (76.1% vs. 74.5%) about equal as men statewide [30]. The prevalence of overweight was about the same with participant obesity (30.4% vs. 24.9%,) and smoking (29.7% vs. 21.0%) higher than for Arizona men of all income levels (45%) [30,31].

The majority of men had several positive food behaviors such as comparing prices, not leaving meat or dairy unrefrigerated for long, and feeding children within 2 hours of waking. However, less than half shopped with a grocery list, planned meals ahead of time, or read the nutrition facts label on foods. Their responses on the Food Behavior Checklist are similar to Arizona women from the same time period and the pre-test results of Nebraska EFNEP adult enrollees [27,32]. Over a third of the men indicated they did not add salt when preparing foods. Notably, the Arizona men’s behavior toward added salt was less frequent than reported in the two other studies [27,32]. Information about selecting “no salt added” canned beans, or dry beans which do not contain salt, may appeal to some low-income men.

Ethnic differences were apparent, with Hispanic men having less food security and less likely to compare food prices or use a grocery list than their peers. Many also indicated their children did not always eat within 2 hours of waking. Hispanic men had more children in their households, and less income on average, yet had equivalent monthly food expenditure to their peers. In a previous study, Hispanic women in metro-Phoenix were more food secure than non-Hispanic white women, but also did not shop with a grocery list nor always feed children promptly after waking in comparison [27]. Since food and storytelling are common themes in Hispanic and black culture, combining behavior change messages in stories and traditional cultural practices may be an appropriate way to convey healthful practices [33].

Shopping with a grocery list has been associated with a higher dietary quality and lower BMI among high-risk adults [34]. Since a high percentage of the low-income non-Hispanic men reported using grocery lists, and “sometimes” or “always” planning meals ahead of time, canned beans may be touted as a versatile option to add to a grocery list and to keep on hand for quick and easy meals.

Most men in this study identified beans as improving nutrition and increasing satiety, but only about a third knew of the well-documented health benefits of beans to lower cholesterol, aid in blood glucose control, and reduce cancer risk [10,12,14]. These health benefit knowledge gaps among low-income men may be mediated by race/ethnicity since more non-white men disagreed that these were known benefits. Similar studies have shown non-Hispanic whites to have greater nutrition knowledge than black, Hispanic and other non-white race/ethnic groups across income levels [35,36].

However, it is difficult to extricate the influence of education from race/ethnicity when known disparities are observed. Other research suggests that those with higher education display greater nutrition knowledge scores [35,36,37]. In response to the same bean health knowledge questions, less-acculturated Hispanic women in Arizona and in Iowa displayed lower nutrition knowledge levels than their bicultural or English dominant peers with more education [26,38]. While nutrition knowledge is a necessary component for healthy food behaviors, it does not appear to be a sufficient driver of food choice on its own [37,38,39]. Doma et al. found in a recent Canadian study of older adults that 98% felt eating beans could improve their health, but only 51.2% reported consuming beans regularly [40].

The low-income men in this study had positive attitudes about dry and canned beans. The majority of Arizona men reported eating beans, liked their taste, and thought it was not difficult to make meals with them. However, these men were not likely to meet the DGA recommended level of 2–3 cups per week since most men reported consuming beans only 2–3 times per month [2]. No difference was found in consumption frequencies by race or ethnicity, which was consistent with equivalent bean consumption frequencies between black and white men in another US study [41]. People had similar positive views of beans yet low rates of consumption in other studies conducted in Mexico, Australia, France, and Canada [42,43,44,45].

Food selection appears to be largely based on taste and cost, with taste cited as the biggest barrier to healthy eating [46,47]. Moreover, consumers have implicit views that “healthy” foods are thought to be less filling and may have an inverse relationship to those that are “enjoyable” [47,48]. Since men in this pilot study perceived beans as satiating and appetizing, and taste is a leading influence on food choice, focusing on taste rather than nutrition may be a beneficial area to pursue in marketing pulses [48,49]. Additional tailored nutrition education messages may highlight preferences of canned beans for white men and dry beans for black and Hispanic men. Monge et al. also found in Mexico that there was a preference among adults for dry, not canned, beans [42]. Likewise, based on these preliminary results, messaging may be tailored to Hispanic men by focusing on aspects of tradition, and to white men by emphasizing economic value.

There are several study limitations including the small sample size for the subgroups of black and Hispanic men. BMIs were calculated from self-reported height and weight and may be slightly lower than if measured directly by researchers [28]. The actual amount of beans consumed was not asked of participants. Results cannot be generalized to populations other than those surveyed.

Despite the cross-sectional convenience sample and small sample size, this study fills a research gap by providing descriptive details on health benefit knowledge, bean consumption patterns and preferences, and attitudes about beans among a diverse sample of low-income men. They are less likely to participate in health behavior research and are an understudied population [49,50]. The men were recruited to answer questions about health and food rather than specifically about beans in an effort to reduce response bias to only those who ate beans. Other topics to address in future research include food environment, selection practices, shopping and preparation behaviors of the household, facilitators and barriers to bean consumption, in what manner beans are consumed (in or outside the home, types of products, cuisine, entrée/side, etc.), and the male perspective on beans as a protein source compared to meat and dairy.

## 5. Conclusions

These findings suggest that low-income men practice some desirable food behaviors, have positive attitudes toward beans, and consume them, but lack knowledge of some health benefits of beans. Leveraging men’s desirable food behaviors, positive views of beans, and increasing awareness of bean health benefits with clear, concise, and achievable messaging could improve health and make an increase in bean consumption more feasible [50].

## Figures and Tables

**Table 1:** Distribution of demographic, household and health risk characteristics of Arizona men utilizing assistance programs by race/ethnicity classifications (N=71).

Characteristics	Total	White 53% (38)	Black 16% (11)	Hispanic 31% (22)
	←mean ± standard deviation (SD)→			
Age in years (±SD) *	41 ± 12.7	45 ± 12.5	42 ± 11.2	35 ± 12.0
Monthly household income $	2352 ± 1719	2757 ± 1893	2225± 1620	1653 ± 1165
Monthly amount spent on food $	329 ± 268	318 ± 197	336 ± 421	343 ± 285
	← % →			
Years of Education **				
11th grade or less	21.1	7.9_a_	18.2_ab_	45.5_b_
High school or equivalent	21.1	18.4_a_	18.2_a_	27.3_a_
Some college or Associates	31.0	34.2_a_	45.5_a_	18.2_a_
Bachelors or Masters	26.8	39.5_a_	18.2_ab_	9.1_b_
**Marital Status**				
Single	39.4	42.1	54.5	27.3
Married/Cohabitating	60.6	57.9	45.5	72.7
Has children under 20 years **	39.4	26.3_a_	18.2_a_	72.7_b_
Born in the US ***	74.3	89.5_a_	81.8_a_	42.9_b_
**Food security category**				
High food security	72.5	71.1	72.7	75.0
Low food security	15.9	18.4	9.1	15.0
Very low food security	11.6	10.5	18.2	10.0
**BMI Category**				
Normal 18.5–24.9	24.6	26.3	30.0	19.0
Overweight 25.0–29.9	44.9	44.7	60.0	38.1
Class I obesity > 30.0–34.9	17.4	15.8	10.0	23.8
Class II+ obesity ≥ 35.0+	13.0	13.2	0	19.0
**Exercise for 30 minutes or more**				
Almost never	23.9	23.7	18.2	27.3
2–4 times per month	28.2	26.3	18.2	36.4
2–3 times per week	23.9	26.3	18.2	22.7
4 or more times per week	23.9	23.7	45.5	13.6
**Cigarette smoking**				
Never smoked	40.8	36.8	45.5	45.5
Quit	21.1	31.6	9.1	9.1
Current smoker	38.0	31.3	45.5	45.5
**Has health care coverage**	54.9	63.2	54.5	40.9
**Routine checkup within**				
Past year	56.3	63.2	54.5	45.5
Past 2 years	22.5	18.4	36.4	22.7
3+ years ago	21.1	18.4	9.1	31.8

*p < 0.05; **p < 0.01; ***p< 0.001. Same subscript letters (a, b, etc.) indicate column proportions that are not significantly different from each other.

**Table 2:** Food behavior checklist (FBC) questionnaire responses among Arizona men utilizing assistance programs by race/ethnicity category (%; N = 71).

For These Questions, Think about How Often You Do These Behaviors.	Total	White 53% (38)	Black 16% (11)	Hispanic 31% (22)
	← % →			
**Food Resource Management**				
1. Compare prices before you buy food? *				
Do not do	8.6	0_a_	18.2_b_	19.0_b_
Seldom	4.3	2.6_a_	9.1_a_	4.8_a_
Sometimes	14.3	13.2_a_	0_a_	23.8_a_
Most of the time/Always	72.9	84.2_a_	72.8_a,b_	52.4_b_
**2. Shop with a grocery list?**				
Do not do	14.3	7.9	27.3	19.0
Seldom	15.7	15.8	18.2	14.3
Sometimes	20.0	13.2	18.2	33.3
Most of the time/Always	50.0	63.2	36.4	33.3
3. Run out of food before end of month? **				
Do not do	22.9	26.3_ab_	0_b_	28.6_a_
Seldom	30.0	31.6_a_	9.1_a_	38.1_a_
Sometimes	40.0	39.5_a_	90.9_b_	14.3_c_
Most of the time/Always	7.1	2.6_a_	0_a,b_	19.0_b_
**Food Safety Behaviors**				
**4. Let meat and dairy foods sit out for >2hrs**				
Do not do or N/A	70.0	76.3	54.5	66.7
Seldom	20.0	13.2	36.4	23.8
Sometimes	8.6	10.5	0	9.5
Most of the time/Always	1.4	0	9.1	0
5. Thaw foods at room temperature *				
Do not do	24.3	21.1_a_	36.4_a_	23.8_a_
Seldom	22.9	34.2_a_	0_b_	14.3_a,b_
Sometimes	28.6	26.3_a_	9.1_a_	42.9_a_
Most of the time/Always	24.3	18.4_a_	54.5_b_	19.0_a_
**Nutrition Practices**				
6. Plan meals ahead of time *				
Do not do	10.0	13.2_a_	9.1_a_	4.8_a_
Seldom	14.3	18.4_a_	18.2_a_	4.8_a_
Sometimes	32.9	23.7_a_	9.1_a_	61.9_b_
Most of the time/Always	42.9	44.7_a_	63.6_a_	28.6_a_
**7. When deciding what to feed your family, how often do you think about healthy food choices?**				
Do not do	7.2	8.1	9.1	4.8
Seldom	8.7	8.1	8.1	9.5
Sometimes	31.9	27.0	27.3	42.9
Most of the time/Always	52.2	56.7	54.6	42.8
**8. Prepare food without adding salt.**				
Do not do	17.1	15.8	0	28.6
Seldom	17.1	10.5	36.4	19.0
Sometimes	24.3	23.7	27.3	23.8
Most of the time/Always	41.4	34.2	27.3	23.8
**9. Use the “Nutrition Facts” or food label to make food choices?**				
Do not do	13.0	10.5	20.0	14.3
Seldom	31.9	18.4	30.0	57.1
Sometimes	23.2	26.3	30.0	14.3
Most of the time/Always	31.9	44.7	20.0	14.3
**10. Children eat within 2 hours of waking up?†**				
Do not do	0	0	0	0
Seldom	8.0	0	0	15.4
Sometimes	4.0	0	0	7.7
Most of the time/Always	88.0	100	100	76.9
Positive food behavior scale (±SD) (Sum of questions 1–9)	3.30 ± 0.85	3.52 ± 0.81	3.10 ± 1.0	3.0 ± 0.76

*p < 0.05; ** *p* < 0.01; †: n = 28 (39%). Same subscript letters indicate column proportions that are not significantly different from each other.

**Table 3:** Bean health benefit knowledge among Arizona men utilizing assistance programs by race/ethnicity category (percentage; N = 71).

Eating Beans Can…	Total	White 53% (38)	Black 16% (11)	Hispanic 31% (22)
	← % →			
1. Improve nutrition *				
Disagree	11.6	0_a_	27.3_b_	23.8_b_
Neutral	29.0	29.7_a_	27.3_a_	28.6_a_
Agree	59.4	70.3_a_	45.5_a_	47.6_a_
**2. Help you feel full**				
Disagree	8.7	8.1	9.1	9.5
Neutral	37.7	37.8	36.4	38.1
Agree	53.6	54.1	54.5	52.4
3. Lower bad cholesterol *				
Disagree	11.6	0_a_	18.2_b_	28.6_b_
Neutral	49.3	51.4_a_	45.5_a_	47.6_a_
Agree	39.1	48.6_a_	36.4_a_	23.8_a_
**4. Lower cancer risk**				
Disagree	13.0	2.7	27.3	23.8
Neutral	56.5	59.5	54.5	52.4
Agree	30.4	37.8	18.2	23.8
5. Control blood sugar *				
Disagree	17.4	5.4_a_	27.3_b_	33.3_b_
Neutral	53.6	56.8_a_	45.5_a_	52.4_a_
Agree	29.0	37.8_a_	27.3_a_	14.3_a_
6. Healthy gastrointestinal tract *				
Disagree	10.1	0_a_	27.3_b_	19.0_b_
Neutral	43.5	45.9_a_	36.4_a_	42.9_a_
Agree	46.4	54.1_a_	36.4_a_	38.1_a_
**7. Help lose weight**				
Disagree	23.2	10.8	36.4	38.1
Neutral	36.2	40.5	36.4	28.6
Agree	40.6	48.6	27.3	33.3
**Summary Scale**				
Knowledge of bean health benefits ** (±SD) (Sum of questions 1–5)	3.25 ± 0.84	3.53 ± 0.60	2.95 ± 1.14	2.90 ± 0.87

*p < 0.05; **p < 0.01. Same subscript letters (e.g. a, b, etc.) indicate column proportions that are not significantly different from each other.

**Table 4:** Attitudes, perceptions, and consumption preferences for beans among low-income Arizona men by race/ethnicity category (N = 71).

Here Are Some Reasons People Have Said Prevent Them from Eating or Cooking Beans. Do You Disagree, or Agree?	Total	White 53% (38)	Black 16% (11)	Hispanic 31% (22)
	← % →			
**1. Difficult to make food with beans**				
Disagree	76.5	64.9	100.0	85.7
Neutral	20.6	29.7	0	14.3
Agree	2.9	5.4	0	0
**2. Family/children will not eat**				
Disagree	54.4	52.8	45.5	61.9
Neutral	36.8	36.1	54.5	28.6
Agree	8.8	11.1	0	9.5
**3. Do not like taste of beans**				
Disagree	72.5	73.0	72.7	71.4
Neutral	14.5	13.5	9.1	19.0
Agree	13.0	13.5	18.2	9.5
**4. Only poor people eat beans**				
Disagree	75.4	73.0	72.7	81.0
Neutral	23.2	27.0	18.2	19.0
Agree	1.4	0.0	9.1	0.0
**5. Beans not eaten by friends**				
Disagree	68.1	62.2	81.8	71.4
Neutral	29.0	32.4	18.2	28.6
Agree	2.9	5.4	0	0
**6. Beans cause intestinal gas**				
Disagree	14.5	8.1	18.2	23.8
Neutral	37.7	43.2	27.3	33.3
Agree	47.8	48.6	54.5	42.9
**7. Dry beans take too long to prepare**				
Disagree	48.5	45.9	50.0	52.4
Neutral	19.1	21.6	30.0	9.5
Agree	32.4	32.4	20.0	38.1
**General bean attitude scale** (μ ± SD) **Summary of 1–5§**	20.0 ± 3.6	19.8 ± 3.7	20.1 ± 2.4	20.9 ± 3.9
**8. Canned beans not true to culture**				
Disagree	71.0	67.6	72.7	76.2
Neutral	24.6	29.7	18.2	19.0
Agree	4.3	2.7	9.1	4.8
**9. Family will not eat canned beans**				
Disagree	61.2	55.6	81.8	60.0
Neutral	26.9	27.8	18.2	30.0
Agree	11.9	16.7	0	10.0
**10. If I cannot cook beans myself will go without**				
Disagree	60.9	62.2	54.5	61.9
Neutral	27.5	27.0	27.3	28.6
Agree	11.6	10.8	18.2	9.5
**11. Canned beans are not healthy**				
Disagree	57.4	62.2	50.0	52.4
Neutral	30.9	27.0	40.0	33.3
Agree	11.8	10.8	10.0	14.3
**12. Canned beans are too expensive**				
Disagree	56.7	56.8	66.7	52.4
Neutral	34.3	35.1	11.1	42.9
Agree	9.0	8.1	22.2	4.8
**13. Canned beans do not taste good**				
Disagree	47.1	55.6	27.3	42.9
Neutral	29.4	25.0	45.5	28.6
Agree	23.5	19.4	27.3	28.6
**14. Canned beans have preservatives**				
Disagree	17.9	13.5	30.0	20.0
Neutral	38.8	48.6	30.0	25.0
Agree	43.3	37.8	40.0	55.0
**Canned bean attitude scale** (μ ± SD) **Summary of 8–13§**	22.6 ± 4.4	23.0 ± 5.1	22.4 ± 3.2	22.0 ± 3.4

**§**: Items reverse coded for summation.

**Table 5:** Bean consumption frequencies, purchasing practices, and preferred characteristics of low-income Arizona men by race/ethnicity (%; N = 71).

Characteristics	Total	White 53% (38)	Black 16% (11)	Hispanic 31% (22)
**Bean consumption frequency**				
Once per month or less	21.1	28.9	18.2	9.1
2–3 times per month	42.3	42.1	45.5	40.9
1–2 times per week	29.6	26.3	36.4	31.8
3+ times per week	7.0	2.6	0	18.1
**Type of bean purchased**				
Both (dry and canned)	47.8	44.4_a_	40.0_a_	57.1_a_
Dry bean only	16.4	5.6_a_	40.0_b_	23.8_b_
Canned bean only	11.9	19.4_a_	0_a_	4.8_a_
Does not buy beans	23.9	30.6_a_	20.0_a_	14.3_a_
**Knows how to prepare dry beans**	81.4	73.7	90.9	90.5
**Frequency someone cooks dry beans in household**				
Once a month or less	40.3	45.9_a_	45.5_a_	26.3_a_
2–3 times per month	31.3	35.1_a_	36.4_a_	21.1_a_
1–2 times per week	17.9	10.8_a_	18.2_a_	31.6_a_
3+ times per week	10.5	8.1_a_	0_a_	17.3_a_
**Of the 60.6% (43/71) who buy dry beans**				
**Preferred packaging**				
Small bags	53.8	66.7	37.5	52.9
Medium bags	34.9	33.3	50.0	29.4
Large bags	9.3	5.6	0	17.6
Loose or in bulk	30.2	33.3	25.0	29.4
**Important selection traits dry**				
Price *	58.1	32.6_a_	4.7_b_	20.9_ab_
Tradition **	39.5	16.7_a_	25.0_a_	70.6_b_
Nutritional value	30.2	38.9	25.0	23.5
Quality	23.3	22.2	25.0	23.5
Taste of beans	20.9	22.2	0	29.4
Brand	25.6	22.2	50.0	17.6
Color of beans	14.0	16.7	12.5	11.8
Cook quickly	14.	11.1	12.5	17.6
Shape	4.7	5.6	0	2.3
**Prefer to buy dry beans from a specific country?**				
No country preference	60.5	50.0	50.0	76.5
Yes, Mexico or Central America	16.3	11.1	12.5	23.5
Yes, USA or other	23.3	38.9	37.5	0
**Of 91.5% (65/71) who buy canned beans**				
**Characteristics of canned beans**				
Price *	58.1	71.8_a_	33.3_ab_	35.3_b_
Taste of beans	43.5	38.5	50.0	52.9
Quality	28.6	35.9	14.3	17.6
Nutritional value	17.7	17.9	33.2	11.8
Tradition—always buy type	17.5	12.5	16.7	29.4
Brand	17.7	20.5	33.3	5.9

*p < 0.05; **p < 0.01. Same subscript letters (e.g., a, b, etc.) indicate column proportions that are not significantly different from each other.
